# What is eHealth (6)? Development of a Conceptual Model for eHealth: Qualitative Study with Key Informants

**DOI:** 10.2196/jmir.8106

**Published:** 2017-10-24

**Authors:** Tim Shaw, Deborah McGregor, Melissa Brunner, Melanie Keep, Anna Janssen, Stewart Barnet

**Affiliations:** ^1^ Research in Implementation Science and eHealth Faculty of Health Sciences University of Sydney Sydney Australia; ^2^ Faculty of Education and Arts University of Newcastle Newcastle Australia

**Keywords:** qualitative research, interview, health care, eHealth, models, theoretical

## Abstract

**Background:**

Despite rapid growth in eHealth research, there remains a lack of consistency in defining and using terms related to eHealth. More widely cited definitions provide broad understanding of eHealth but lack sufficient conceptual clarity to operationalize eHealth and enable its implementation in health care practice, research, education, and policy. Definitions that are more detailed are often context or discipline specific, limiting ease of translation of these definitions across the breadth of eHealth perspectives and situations. A conceptual model of eHealth that adequately captures its complexity and potential overlaps is required. This model must also be sufficiently detailed to enable eHealth operationalization and hypothesis testing.

**Objective:**

This study aimed to develop a conceptual practice-based model of eHealth to support health professionals in applying eHealth to their particular professional or discipline contexts.

**Methods:**

We conducted semistructured interviews with key informants (N=25) from organizations involved in health care delivery, research, education, practice, governance, and policy to explore their perspectives on and experiences with eHealth. We used purposeful sampling for maximum diversity. Interviews were coded and thematically analyzed for emergent domains.

**Results:**

Thematic analyses revealed 3 prominent but overlapping domains of eHealth: (1) health in our hands (using eHealth technologies to monitor, track, and inform health), (2) interacting for health (using digital technologies to enable health communication among practitioners and between health professionals and clients or patients), and (3) data enabling health (collecting, managing, and using health data). These domains formed a model of eHealth that addresses the need for clear definitions and a taxonomy of eHealth while acknowledging the fluidity of this area and the strengths of initiatives that span multiple eHealth domains.

**Conclusions:**

This model extends current understanding of eHealth by providing clearly defined domains of eHealth while highlighting the benefits of using digital technologies in ways that cross several domains. It provides the depth of perspectives and examples of eHealth use that are lacking in previous research. On the basis of this model, we suggest that eHealth initiatives that are most impactful would include elements from all 3 domains.

## Introduction

Despite the growth in eHealth research, there remains a lack of consistency in the use of the term and little consensus on a taxonomy of eHealth technologies [1]. The term *eHealth* has been used to describe a broad range of digital technologies and interventions used by a variety of stakeholders across diverse settings [2-6]. As far back as 2005, a total of 51 unique definitions for eHealth were identified in a systematic review of published definitions of this term [7]. At that time (and to date), the most cited definition was Eysenbach’s [3]:

e-health is an emerging field in the intersection of medical informatics, public health and business, referring to health services and information delivered or enhanced through the Internet and related technologies. In a broader sense, the term characterizes not only a technical development, but also a state-of-mind, a way of thinking, an attitude, and a commitment for networked, global thinking, to improve health care locally, regionally, and worldwide by using information and communication technology.

Although helpful for understanding the broader context of eHealth, definitions can provide limited guidance on the functions and tools captured by the term. Subsequent to publication of this definition (which remains the most cited) [3], Boogerd et al [8] highlighted the evolution of eHealth in clinical practice and queried the need for a literature review to determine an updated definition and creation of a taxonomy for the field. Noted were the increasingly common language but inconsistent use of terms, such as mHealth, telehealth, and telecare, as well as emergent labels, such as medicine 2.0 [9]. In the absence of clarity, terms will continue to be used inconsistently and interchangeably, to the detriment of opportunities for shared discourse and eHealth implementation.

To understand the landscape of eHealth, literature reviews have been used to identify commonly used definitions [7,10] and key components of these definitions [6]. Consistently, these studies have shown that eHealth definitions are varied and have little or no operational clarity. In an attempt to address these limitations, Reed [11] conducted a concept analysis of eHealth in nursing and developed case examples of eHealth, explicitly demonstrating how eHealth is applied and the attributes of eHealth users. Focusing on telemedicine, researchers have developed taxonomies that outlined the elements of telemedicine and the relationships between them [12,13]. These studies provided some clarity around eHealth in particular contexts, and their specificity enables eHealth to be operationalized. On the other hand, this focus reduces the applicability of the case examples and taxonomies to other health contexts (including different clinical disciplines and nonacute care) or applications of eHealth. Building on these studies, further work is required to identify a model of eHealth that is operational and applicable at each step along the health process (from prevention to acute care and long-term management).

Methodologically, literature reviews provide a summary of published definitions, often with a time delay due to publication processes for the original articles and review papers. They capture frequencies and common elements or themes [7] while potentially losing the depth of definitions that are integral for understanding how concepts might be operationalized. Qualitative research methods, on the other hand, are well suited to the discovery of emergent concepts or determining the meaning of a phenomenon [14]. Multiple informant perspectives can generate a valuable snapshot that captures a realistic and current representation of the studied landscape. The diversity of participant perspectives, understandings, and implementation examples are represented in this approach. A snapshot such as this can be extremely valuable in a rapidly evolving environment such as eHealth.

This study aimed to construct a conceptual practice-based model for eHealth by interviewing eHealth practitioners and scholars. This model would enable health professionals in a range of contexts to apply the different components of eHealth to their own practice through shared understanding of eHealth. With an emphasis on operationalizing eHealth, this practical model will serve as a foundation for eHealth innovation, practice, research, education, and policy.

## Methods

### Design

We undertook an exploratory thematic analysis of participant interviews [15,16] to capture a multitude of individual and contextually distinct perspectives across an extended time frame. Since group dynamics and interactions were not the focus of this study, we conducted interviews instead of focus groups. We conducted semistructured interviews for the primary purpose of producing a massive open online course (MOOC) on the topic of eHealth, with timing of interviews dependent on participant availability. A MOOC is an open access Web-based learning resource aimed at large-scale global participation. This study reports on the interview data and analysis using the consolidated criteria for reporting qualitative research [17]. We used the results to inform the development of an interdisciplinary conceptual model upon which the framework of the final eHealth MOOC curriculum was based.

### Setting and Participants

The study was conducted at The University of Sydney. We used purposeful sampling to recruit key informants (N=25) from diverse professional contexts. Inclusion criteria were that they have significant expertise or vested interests in eHealth research, education, practice, or policy. This included potential participants with key strategic or influential positions. A list of potential participants was generated by investigators (TS, DM, MB, and SB), identified via known eHealth networks and identified by the research team from recent eHealth-related conference proceedings. A research officer invited participants via email. Participation was completely voluntary, and we obtained informed consent from all participants.

**Semistructured interview guide.**

1. What does eHealth mean to you?
2. What impact do you think it’s having on health care currently, both personally and professionally?
3. What impact do you see it might have over the next 5 years?
4. How do you feel it might contribute to the quality or safety of your own health care or your work as a health professional?
5. What eHealth technologies are being used in your field?

### Data Collection

We conducted face-to-face, semistructured interviews, in a location negotiated with each participant, using a semistructured interview guide (Textbox 1). Interviews were audio- and videorecorded by the University’s audiovisual staff. Interview questions were designed to encourage open exploration of conceptual understandings and definitions of eHealth, as well as to capture current practice applications of eHealth technologies. The interview guide was developed by the research investigators, informed by previous research [8,18,19], and ratified by members of the Interdisciplinary eHealth Advancement and Research Team (IeHART) at The University of Sydney.

### Data Analysis

Interviews were transcribed verbatim and thematically analyzed [20]. Analysis was conducted by 2 authors (DM and MB), and all transcripts were continuously read to obtain a good overall sense of the material prior to open coding, where code words were assigned to specific segments of text. Line-by-line coding ensured full inclusion of all possible data. We grouped codes by related themes and subthemes, which we systematically refined to reduce redundancy and emphasize prominent groupings. We conducted constant comparative analysis with iterative discussion of emerging and final domains and subcategories. The coding process continued until saturation; that is, until no new themes emerged. During analysis, we highlighted illustrative quotes and grouped them by domains. We used the Delphi method [21,22] to refine the model over multiple iterations, allowing for systematic consideration of the breadth, complexity, and overlapping nature of eHealth technologies and applications, with consensus reached when all investigators came to a majority agreement.

### Ethics

We obtained ethics approval from The University of Sydney Human Research Ethics Committee prior to study commencement (Protocol No. 2014/1017).

## Results

We conducted interviews between August 2015 and April 2016. Interviews ranged from 9 minutes to 95 minutes (mean 28 minutes, median 19.07 minutes). Variations in interview length were predominantly due to the availability or unique contributions of the participants, with some participants contributing detailed examples of eHealth in research or clinical practice.

### Participant Demographics

A total of 25 key informants participated in the interviews. Participants’ professional specialties and work contexts were specified by their primary place of employment, appointment, and key duties at the time of the study (Table 1). Some participants held senior roles in their organizations, such as faculty deans, academic directors, senior administrators, or chief executive officers. Other participants were health care clinicians, researchers, academics, and PhD candidates. All participants were primarily located in Australia, except for 1 participant who was located in the United States. Despite the large representation of the Australian health context, several participants were involved in international collaborations and offered examples of international eHealth implementation and contexts.

### Views on the Definition and Scope of eHealth

The breadth of participants’ definitions of eHealth varied widely. The range of definitions and examples they provided emphasize the need for a framework that encapsulates both the current eHealth landscape and eHealth practice into the future. Responses to “What does eHealth mean to you?” ranged from the traditional (representative of current literature):

eHealth is a way to incorporate technology into health care to promote health and well-being. It can be as simple as using some form of technology to self-monitor your activity, communicate with different people about health and health conditions, coordinating care within the health system, and actively using technology to provide intervention.

to those that could be considered progressive:

eHealth is so pervasive now[a]days in health research and the implementation of and practice of health that it’s almost like the oxygen [of health]...It involves the collection, the management, the analysis, and the communication of all health-related data. That spans individuals, individual patients, all the way through to entire health care systems.

or possibly contentious:

You know eHealth is really old fashioned? Nobody talks about eHealth anymore. Electronic health—everything's electronic! The devices, everything! We’re talking about digital health, digitizing health, not eHealth.

and thought provoking:

eHealth means the ability to dial a doctor from home and the interconnectedness of all of our medical details—it’s the future we were promised from sci-fi!

**Table 1:** Demographic characteristics of participants (N=25).

Characteristics		n (%)
Male		13 (52)
Female		12 (48)
**Professional specialties**		
	Senior health administration/executive management	4 (16)
	Psychology	3 (12)
	Exercise and movement science/coaching	2 (8)
	Information technology	2 (8)
	Physiotherapy	2 (8)
	Speech pathology	2 (8)
	Clinical health informatics	1 (4)
	Data science	1 (4)
	Digital gaming	1 (4)
	Engineering	1 (4)
	Genetics/genomics	1 (4)
	Medical radiation science	1 (4)
	Metabolic health and chronic disease	1 (4)
	Neurology	1 (4)
	Patient-based care	1 (4)
	Patient-reported outcomes	1 (4)
**Professional contexts**^a^		
	University (education and research)	20 (80)
	Health care delivery (hospital, community, private practice, Web based)	9 (36)
	Governing department of health (state or federal)	3 (12)

^a^Includes participants with multiple professional contexts.

With a global focus on the implementation of electronic records, responses suggested that eHealth has, at times, been considered synonymous with electronic health records and electronic medical records. However, as exemplified by participant descriptions, the scope of eHealth is much broader. Participant examples of digital technologies in health included mobile devices, software apps, wearables, social media, the Internet, Web-based portals and programs, specific software, information management systems, data warehouses, digital gaming, and virtual reality. Practical examples of eHealth technologies encompassed remote service provision, health monitoring, care planning and coordination, communication, information storage and exchange, precision and predictive health care, professional support and development, and consumer empowerment.

Several participants spoke of the intended aims of eHealth, including enabling best care, ensuring safety and quality, enhancing existing services, improving access, connecting points of care, and supporting human health in general. One participant expressed that:

To me, eHealth is about the use of new technologies to create new models of care. “E” to me is enabling, how technologies are enabling new delivery, health services, health efficiencies, and overall improvement to health quality.

Participants emphasized that the focus should not be placed on the technologies but on the potential benefits and improvements that they afford; for example:

It’s not about the technology, it’s about using tools to do what we do, better, faster, safer, more patient-centric, broadening horizons...that’s eHealth.

Participants also highlighted that eHealth implementations need not be overt, as exemplified in the following quote:

The patient probably won’t even notice eHealth because that just means that we have the right tools in the right place being able to be used by the patients and the clinicians, and really that just supports the delivery of the best possible care.

Analysis of stakeholder groups confirmed that eHealth stakeholders are multidisciplinary, spanning medical and allied health professionals, and inclusive of professionals in the social sciences and humanities. Also included are professional groups perhaps not typically thought to be associated with health care, such as professionals in engineering, information technology, business, and economics. Health consumers were an emphasized stakeholder group, encompassing broad demographics. Multiple examples were provided of implementations involving young adolescents, in particular associated with access to mental health services. eHealth interventions were thought to be particularly relevant to the younger population due to their digital competence and their motivation for engagement with digital platforms, including social networking sites. Examples also highlighted the engagement of the older population with eHealth to improve lifestyle behaviors and manage chronic health issues. Caregivers were another noted stakeholder group. Examples of consumer engagement with eHealth were provided right across the health and wellness spectrum, from monitoring and maintaining wellness on an independent basis, through to their engagement with health services and health care providers spanning primary, secondary, and tertiary care.

### Domains

Analysis of the interview data revealed 3 dominant eHealth domains: (1) health in our hands, (2) interacting for health, and (3) data enabling health, with each encompassing several subcategories (Table 2).

#### Domain 1: Health in Our Hands

Analysis revealed repeated reference to mobile devices (eg, smartphones, tablets, and clinical devices), mobile sensors and wearables, apps, social media, and online information. Referring to the personal, accessible, and mobile nature of eHealth technologies that enable access to health information as and when needed, this domain is named “Health in our hands.” One participant, a university researcher, summarized several aspects of this domain as follows:

I put my pedometer or my fitness app and it tells me how many steps I’ve taken, how many hours I slept. I have a sore throat when I go to Google and gives me information from a huge variety of sources, or I go onto an online support program and I again get access to the stories and the experiences and the recommendations and advice of all sorts of people who have gone through what I’m going through.

Participants emphasized how this area is fueled by consumer enthusiasm for gadgets and personal health informatics. The “quantified self” movement and exponential growth in the mobile health technology market has led to increased recording and monitoring of personal health data [23]. Participants noted, coupled with increased consumer health literacy, the growth of a population invested in their own health and well-being. Within this domain, participants listed multiple benefits in terms of improving access, empowering consumers, and facilitating behavior change.

##### Health, Not Just Health Care—Solutions for Health and Well-Being

Within this subcategory, the relevance of and potential for eHealth supporting health and wellness, as distinct from health care, was emphasized. Participants highlighted that managing one’s health and participating in health care transcend interactions with health care professionals or health services. It was emphasized that health and well-being happens on a day-to-day basis and that most people spend very little time with a health care provider or service each year. Rather, they spend much more time and effort self-monitoring and self-caring to maintain health and wellness. One participant, a university researcher, asserted that a shift in mentality is required to:

...stop thinking about health and health care as synonymous things and eHealth will encourage that. It’s not about fixing people when they’re unwell; it’s about making sure people are well for as long as they can be.

Participants acknowledged increases in age-related illnesses and chronic conditions, and the positive role eHealth technologies can play in managing the impact. Multiple examples were provided of eHealth tools being integrated into everyday life, assisting individuals to remain well, out of the health care system, and to participate in life to their full potential. Participants spoke of how increasing consideration is being given to how digital health technologies can be integrated into everyday settings, such as homes, schools, workplaces, and the community.

**Table 2:** eHealth domains and subcategories.

Domain		Subcategories
1	Health in our hands: the use of eHealth technologies to monitor, track, and inform health	Health, not just health care
		Consumer-driven and -controlled health
		Health via social media and the Internet
2	Interacting for health: the use of technologies to communicate between stakeholders in health	Connecting for real-time health
		Social discourses and storytelling
		New ways of interacting to personalize care
		Supporting health professionals
3	Data enabling health: the collection, management, and use of health data sources	Data management systems and data repositories
		Data for precision health
		Data enabling quality

One participant, a university researcher, referred to “positive computing,” how technologies are being designed to specifically support psychological well-being and human flourishing [24]. Examples were provided outlining how apps are being used to provide users with positive reinforcement through a meaningful text message, a personal image, or quantification of a self-generated goal for compliance with a healthy behavior, such as drinking water or using a preventive inhaler. Within mental health contexts, examples included supporting individuals at risk of substance abuse or self-harm. These included the use of mobile devices, apps, and global positioning system coordinates to monitor for specified trigger events. If a trigger event occurs, the user is provided with an immediate response on the device, and a nominated person (such as a partner or professional counsellor or psychologist) is alerted via short message service (SMS). A health executive expressed that:

In the prevention end, it’s going to be about consumers owning their own health, and the devices that are available will be there to support them to do that.

##### Consumer-Driven and -Controlled Health

A recurring theme was that this is the “dawn of consumer-driven health care.” Patient centeredness is a well-recognized factor for high-quality health care [25], and participants acknowledged that consumers are increasingly taking control of or playing a more active role in their health. There was a strong sense that innovations in eHealth are being driven by demand for consumer-oriented solutions. However, it was acknowledged that health care remains slower than other customer-focused industries, such as banking and finance, in integrating technology with service users.

Commonly cited examples of consumer-driven and -controlled health included access to and control of their personal electronic health records, such as the My Health Record in Australia [26]. OpenNotes in the United States [27] was another cited example, which gives consumers access to the clinical notes captured in their medical record. Consumer access and control is thought to empower the consumer to take greater control of their health, foster involvement in decision making [28], and promote a more equal relationship with their health care [29]. This was reinforced by a health executive who asserted that:

Patients are really the experts here, they’re the people who know themselves best, and they have a great opportunity to be able to contribute information and to really manage that information.

Another health executive reinforced the value of patient access to health records in terms of quality and safety:

I don’t think anybody cares more about the information about a patient than the patient themselves. So if we can show them that information and they can be part of the discussions about that information, that is inherently a very powerful safety and quality measure.

Participants noted that health consumers increasingly have access to information that affects their choices about the care they receive. This includes not only access to evidence-based online health information, but also ratings of health professionals and experiences of care [30-32].

##### Health Via Social Media and the Internet

There was particular emphasis on the role of social media in health and well-being within this domain. Participants identified a range of social media platforms that were driving online health communities, including blogs, such as WordPress; collaborative projects, such as Wikipedia; social networking sites, such as Facebook and Twitter; content communities, such as YouTube; virtual social worlds, such as Second Life; and social online games [33]. There was particular focus on consumer use of social media to obtain information and connect with other people with similar experiences or a common diagnosis. Similarly, health professionals noted their use of social networking platforms, like Twitter, to access professional information and networks.

Participants discussed how health consumers are using social media and the Internet to check symptoms, gather and clarify information, compare options, and potentially to self-treat, as captured in the following quote:

I think we will go to our [general practitioner] much more knowledgeable, knowing what our problem is, and we will be expecting to have choices offered to us so that we can make reasonable decisions about where we should be going next.

One participant described the development of a patient portal that tracks their activity to capture what information they are searching for online and displays this on a dashboard for the care provider. The participant, a researcher and clinician, explained how the portal aims to enrich the encounter between the patient and the provider and encourage collaborative decision making:

So it’s another window into what that patient’s state is...What are questions that’s important to that patient? So rather than spending that time procuring that information in the clinical encounter, they have that available to them.

With increased access to computers at the bedside, mobile devices, and the Internet, clinicians noted how point-of-care tools provide just-in-time access to evidence-based information to support clinical decision making. A clinician expressed that:

...you have so much access to information in your pocket, when you’re doing things like recharting medications for a patient or explaining something, to be able to pull out your phone and look it up and get the answer right there on the spot.

#### Domain 2: Interacting for Health

Participants discussed the impact of eHealth on health communication. They emphasized that, although this domain may have been traditionally dominated by teleconferencing and videoconferencing, the field increasingly includes a wide range of synchronous and asynchronous communication tools, such as SMS and push notifications from apps, patient storytelling through dedicated portals and social media platforms, and via virtual or simulated therapy tools. Transcript analysis revealed that eHealth plays a role in multiple communicative interactions, including provider to consumer, provider to provider, and consumer to consumer. Participants acknowledged that eHealth is providing new ways of interacting and, as such, enabling new models of care.

##### Connecting for Real-Time Health

One participant argued that, despite advances in technology, we have not yet replaced, nor perhaps should we ever replace, the need for health professionals and consumers to communicate with one another. Participants referred to multiple examples of digital health services, including virtual consultations, telehealth clinics, and Web-based group forums, using a variety of telecommunications and Web-based conferencing software to connect for real-time interactions. Examples included a physiotherapist whose Web-based business is delivering interventions for musculoskeletal injuries to clients worldwide and an exercise physiologist who provides remote consultations for cancer rehabilitation and supportive care services. The following quote from a clinician captured how such digital health services give the consumer an advantage:

The Skype or videoconferencing service was really convenient. He could book in via his Outlook or email system that he would use for work, and treat this like he would a normal appointment, and reduce the time from 2-3 hours with driving to half an hour of purposeful engagement with that health professional.

A neurologist provided the following example of how they are connecting health professionals and patients between satellite and specialist centers to deliver an acute care telestroke service:

The ceiling camera allows us to communicate with the patient, with the relatives of the patient, and with the nurses, and with the help of the nurses or the emergency department physicians, we are capable to perform an assessment of the patient that just came into the [satellite] department.

In another example, an emergency department physician explained how connecting in these ways increased opportunities to accurately triage a critically ill patient at a distance, limit unnecessary transfer to a central facility, and start advanced treatments early. In terms of outcomes, he noted that these interventions often meant that the patient could be transferred with lower levels of support and arrive at the central facility in a more stable condition.

In each of the provided examples, improved access by bridging temporal and geographical limits was cited as a distinct advantage of eHealth, as exemplified in the following quote:

eHealth, to me, means delivering appropriate care to patients that would have otherwise missed out because of location.

##### Social Discourses and Storytelling

Participants discussed the opportunities afforded by social media for discourses about health that may not be possible in face-to-face conversations. They noted how it allows for professional and peer interactions that can be either overt or anonymous. One participant described how a moderated, anonymous, and secure online mental health platform for young adolescents provides a community of support that empowers participants to maintain healthy behaviors.

Social media was considered highly amenable to consumer storytelling. As highlighted by a psychology researcher:

Social media reminds us that our stories are important and our voices need to be heard.

It was acknowledged that, while perhaps not medically trained, consumers have experiential knowledge that is very powerful in recovery and motivating others.

##### New Ways of Interacting to Personalize Care

One physiotherapist asserted that, for health care services to be successful, now and into the future, they have to “effectively harness that intersection between automation and personalization.” Clinicians acknowledged the increasing integration of digital tools into practice to customize services, including the prescription of tailored therapy and rehabilitation programs. For example, participants from physiotherapy and exercise physiology outlined the use of apps to tailor therapy programs with personalized advice, education materials, and high-quality video demonstrations. They explained how digital tools are providing key interventions benefits and new service choices for clients, such as in-home therapy involving remote consultation, monitoring, and program adjustment. In other examples, participants highlighted how apps are helping consumers to alert health care professionals to changes in their condition, including for in-home renal patients who log their own health data and self-evaluations of their current wellness status to be monitored by service-based clinicians.

A speech pathologist described how Twitter and other social media platforms are being used in communication rehabilitation for clients who have a traumatic brain injury. She described how communicating via social media reduces literacy demands, allows time for message composition and message processing, and provides options for photos or hyperlinks, which may be of significant advantage for someone with a communication impairment. These individuals may then “use social media so that they can communicate in what is considered a normal everyday activity.”

##### Supporting Health Professionals

Participants acknowledged that eHealth technologies have a significant role in supporting health professional interactions for interprofessional collaboration, remote mentoring, and professional support of new, generalist, or isolated providers. In particular, participants emphasized the advantage of eHealth-enabled information exchange and conferencing for collaborative case reviews and discussions that support diagnostic and therapeutic decision making. Examples included models of distributed professional collaboration, such as multidisciplinary cancer care team case reviews.

Participants highlighted how digital technologies are increasingly embedding relevant information and just-in-time learning episodes into routine workflows. Examples included alerts within electronic record systems and information delivered via clinical decision support tools at the point of care. One participant discussed a targeted professional development program that provides learners with short case-based learning scenarios via email or app. They outlined how the use of routinely collected clinical data is starting to inform more adaptive or tailored professional development activities directly related to their actual clinical encounters. The university researcher and clinician explained:

So you have to contrast that to what we’ve done traditionally, which is just sort of “one size fits all;” not customized to the patient, to the clinical encounter; not in the workflow...This is all an effort to now have it embedded in the clinical environment.

#### Domain 3: Data Enabling Health

Participants reported being in the middle of a health care reformation, whereby access to and use of vast amounts of health-related data are being realized. This domain encompasses the collection, management, analysis, and application of health data, including the design and implementation of technologies that provide new and expanded forms of knowledge about ourselves as individuals, our community, and the population as a whole.

##### Data Management Systems and Data Repositories

A prominent theme under this domain was the emphasis on the role of electronic medical records and electronic health records in the collection, storage, and communication of health data, and in particular, routine clinical data. One health executive described the function of electronic records as both the central repository of health information and a communication tool that enables the sharing of information across a network of providers. Yet, despite the intense implementation efforts worldwide, participants acknowledged that electronic records are, as yet, not as seamless as perhaps expected by both providers and consumers. Participants spoke at length about implementation efforts dedicated to overcoming challenges of privacy and security, connectivity and integration of data across silos of information and various provider systems, completeness and quality of data, and development of commonly agreed-upon information standards.

While conventional electronic record systems dominated discussions, a unique example of a consumer-owned and -controlled data management system provided insight into future possibilities. The university researcher referred to the concept of the “unpatient,” where people are the custodians of their data and their personal health records, not the government or health providers. In this example, personal health data, including familial history, specialist reports, pathology records, lifestyle data, and even genomic sequencing, were recorded and maintained on a personal mobile device. In the future, the participant would like to see electronic health data from personal data management systems shared securely with nominated people, including medical professionals and researchers.

##### Data for Precision Health

Participants acknowledged the opportunities and challenges associated with the growing swathes of data, including the abundance of routine clinical data and emergence of new forms of consumer-generated data, such as the data generated from personal devices and monitors and patient-reported outcome measures. They acknowledged that, while data from personal trackers, such as steps taken and calories eaten, provide useful information about lifestyle, as yet, the data don’t capture the detail necessary to predict and personalize how we deliver health care. One university researcher described how making sense of data will further our understanding of the complex relationship between biology and environment to better inform how health care can be delivered in a personalized or “precise” way.

Participants also noted the exponential growth in research activities involving large omics datasets. They agreed that there is tremendous potential for the abundance of health-related data to have significant impact on health and wellness of an individual, cohorts of individuals, and the population as a whole. They expressed enthusiasm for the linkage and integration of various data sources contained within electronic health records and other data repositories, as captured in the following quote:

Working in genetics, I think the idea of having a full set of data is where things become important. You can have lots and lots of data and you can have these huge troves within the genome, but if it’s not connected to the clinical side or the phenotype, it really becomes quite useless.

Participants frequently referred to the role of big data analytics and the need for sophisticated procedures to manage data into a form that is tractable for designing personalized interventions, maintaining health, and predicting and preventing disease. They suggested that one of the most exciting things about eHealth is the potential to enable data-driven care. Participants spoke of the increasing potential of data analytics to determine an individual’s likely health trajectory and inform diagnostics and clinical decision making. Clinician participants reinforced the desire to have linked datasets and real-time clinical decision support tools that interrogate data sources. Ultimately, they look forward to the day when complex data analytics provide them with the most appropriate information and relevant options to guide best practice and personalize care for their clients.

##### Data Enabling Quality

Participants emphasized that eHealth is contributing to safer and higher-quality health care. One health executive noted the potential for eHealth technologies to reduce harm, especially in situations that are subject to human error. Electronic medication systems, for example, were noted to reduce some of the risks associated with dispensing pharmaceuticals. Other examples included the emergence of expert guided computer intelligence systems that integrate expert knowledge and reasoning with best evidence, such as Dr Watson by IBM.

Participants discussed how data analytics can provide information about the quality of the health care experience and enable more informed decisions about quality improvement priorities. They discussed how electronic record systems have facilitated access to routine data for the purposes of quality indicator implementation, performance feedback, and quality improvement. Discussions included the potential for data visualization tools to inform performance and behavior change for individual clinicians, clinical teams, and whole organizations.

## Discussion

### Principal Findings

**Figure 1:**
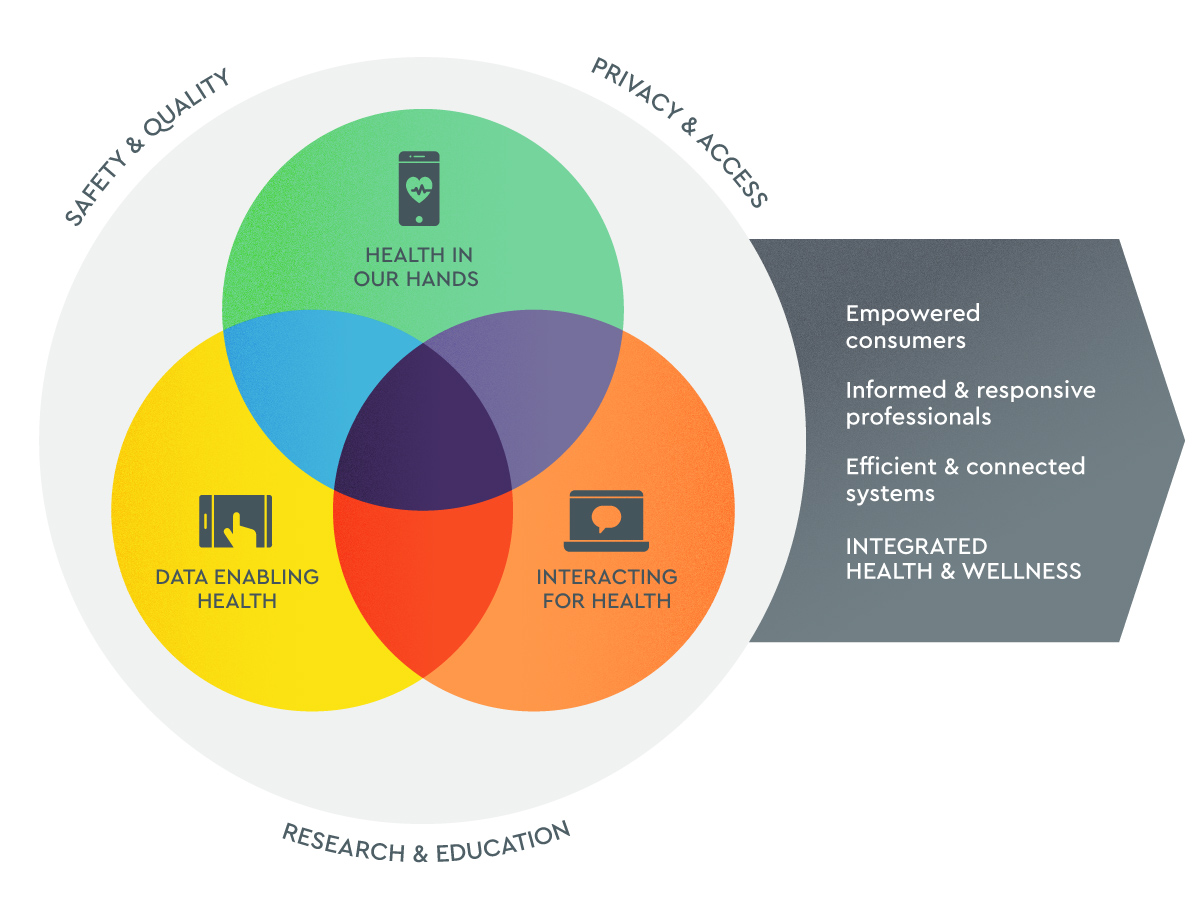
A conceptual model for eHealth.

We interviewed eHealth practitioners, scholars, and policy influencers to develop a model for conceptualizing eHealth (Figure 1). This model responds to calls in the recent literature for an updated, operationalizable definition of eHealth and a taxonomy of eHealth technologies [8]. Informed by the dominant themes emerging from the qualitative interviews, the model consists of 3 overlapping domains: (1) health in our hands, (2) interacting for health, and (3) data enabling health. Separately, these domains describe, respectively, the use of digital technologies to monitor, track, and inform; the use of digital technologies to facilitate communicative encounters between health stakeholders; and the use of data to improve health and health services.

### The Overlapping Nature of eHealth

A distinctive feature of eHealth, however, is its fluid boundaries. Previous research has identified numerous overlapping definitions for eHealth. This is, in part, a key limitation of studying eHealth, but also a distinctive feature of the field. Classifying telemedicine along 3 dimensions (type of technology; perspective of the individual, such as client or practitioner; and context in which eHealth is applied), Tulu et al [13] demonstrated the necessity of overlap between different domains of eHealth. For example, a social networking site used to provide social support for consumers sharing a diagnosis can be categorized under domain 1, health in our hands, and domain 2, interacting for health. The overlapping nature of our model acknowledges the complexity of eHealth while providing a practical way of understanding how eHealth is perceived and implemented.

Of particular importance is the role of overlap among these domains for guiding the development of highly impactful innovations. In particular, where all 3 domains overlap is the optimum point that integrates health data for enhancing interactions and communications so as to empower consumers to be active in their health and health care. The model provides a conceptual framework that can assist individuals and organizations in developing and integrating eHealth initiatives and transforming current models of care. We propose that interventions incorporating multiple domains have the greatest potential impact. For example, the developer of an app targeting self-management of a chronic health condition will consider how the user interacts with the technology to monitor or manage their condition (health in our hands); how it provides opportunities for communication and interactions with caregivers, peers, or professionals for monitoring, coaching, or support (interacting for health); and how gathered data are stored, managed, and analyzed for immediate decision support and, increasingly, personalized and precision health care (data enabling health).

One participant exemplified this in a mental health program they described that provides an online assessment of a program participant’s mental health status using validated tools, automatically recommends reviewed and evidence-based apps, makes an appointment for the participant with a virtual or face-to-face consultation if required, and stores all data in a format that can be included in a health record for future review. Similarly, in the case of an individual health professional, the model provides a framework for evaluation and decision making regarding digital technologies being considered for implementation or prescription. The health practitioner can use the model to guide how they effectively implement eHealth into their practice. For instance, when looking at recommending apps such as those designed to monitor blood sugars, the health professional needs to understand how the app will be used collaboratively with their patient, how this may affect their collaboration, and how any data may usefully be stored and used in consultations. We assert that the future of eHealth lies with technologies that incorporate all 3 of the highlighted domains.

At The University of Sydney, this model has been used to underpin research and education in eHealth and has formed the basis for curriculum development in allied health education.

To the best of our knowledge, no other model or conceptual framework exists that provides a practical guide for both the development of eHealth resources and the application of these resources into an individual’s practice. The model aligns with Black et al [1], who described eHealth technologies as having 3 main overlapping functions: (1) to enable the storage, retrieval, and transmission of data; (2) to support clinical decision making; and (3) to facilitate remote care.

### Limitations and Future Research

Strengths of the study include the breadth of experience and clinical disciplines possessed by the stakeholders and experts whom we interviewed. The interview methodology also allowed for greater depth of understanding about how eHealth is conceptualized and implemented, and included examples that previous literature reviews did not capture. Limitations of this study include that the key informant interviews did not include health consumers. Future research could test the assumption that multidomain eHealth initiatives would be more impactful. Further validation through interviews with health consumers would also strengthen the model.

The development of this model provides a framework to guide discussion and development of eHealth in practice in a rapidly evolving market. For health professionals, educators, researchers, and consumers, this model may help to inform how eHealth can facilitate coordinated care and wellness into the future. For funders of health care, such as governments and health insurers, it provides a framework that can be used to maximize the return of investment on the development of tools to support health and wellness. This includes the development of mobile ecosystems that integrate the 3 domains of this model into easy-to-access and integrated “one-stop shops” for supporting health and wellness and positive behaviors. This model extends current understanding of eHealth by providing clearly defined domains of eHealth while highlighting the benefits of using digital technologies in ways that cross several domains. It is clear from this model that there is significant overlap between aspects of these eHealth domains, and it is important not to draw boundaries around each of them too tightly. Perhaps the greatest strength of this model is identifying the “sweet spot” where the domains coalesce to provide the ideal integration of informed consumers, proactive health professionals, and a responsive health system. The model may enable awareness of how eHealth can empower professionals and health consumers alike to be more active participants in ongoing health and well-being management. It may also facilitate greater clinical and organizational understanding of the application of eHealth resources into practice for better outcomes for all. At this point in time, the model ultimately provides a rich snapshot description of the overlapping nature and broad scope of eHealth in a health care landscape that continues to transform the way in which we view health and well-being.

## Abbreviations

IeHART: Interdisciplinary eHealth Advancement and Research Team
MOOC: massive open online course
SMS: short message service
